# Alteration in Rab11‐mediated endocytic trafficking of LDL receptor contributes to angiotensin II‐induced cholesterol accumulation and injury in podocytes

**DOI:** 10.1111/cpr.13229

**Published:** 2022-05-14

**Authors:** Jijia Hu, Zijing Zhu, Zhaowei Chen, Qian Yang, Wei Liang, Guohua Ding

**Affiliations:** ^1^ Division of Nephrology Renmin Hospital of Wuhan University Wuhan Hubei China; ^2^ Nephrology and Urology Research Institute of Wuhan University Wuhan Hubei China

## Abstract

**Objectives:**

Exposure of podocytes to angiotensin II (Ang II) enhances the abundance of the cell surface glycoprotein, low‐density lipoprotein receptor (LDLR) and promotes significant changes in the cellular cholesterol content. Recent investigation provides evidence that the small GTPase Rab11 is involved in the regulation of LDLR, but the exact mechanisms remain unknown. In this study, the role of Rab11 in post‐transcriptional regulation of LDLR was evaluated to investigate potential mechanisms of podocyte cholesterol dysregulation in chronic kidney disease.

**Materials and Methods:**

Cholesterol content, LDLR and Rab11 expression were assessed in podocytes from Ang II‐infused mice. In vitro, the intracellular localization of LDLR was detected under different conditions. Rab11 expression was modulated and we then explored the effect of anti‐lipid cytotoxicity by detecting LDLR expression and trafficking, cholesterol content and apoptosis in podocytes.

**Results:**

Cholesterol accumulation, upregulated expression of LDLR and Rab11 were discovered in podocytes from Ang II‐infused mice. Ang II enhanced the co‐precipitation of LDLR with Rab11 and accelerated the endocytic recycling of LDLR to the plasma membrane. Additionally, silencing Rab11 promoted lysosomal degradation of LDLR and alleviated Ang II‐induced cholesterol accumulation and apoptosis in podocytes. Conversely, overexpression of Rab11 or inhibition of lysosomal degradation up‐regulated the abundance of LDLR and aggravated podocyte cholesterol deposition.

**Conclusions:**

Rab11 triggers the endocytic trafficking and recycling of LDLR; overactivation of this pathway contributes to Ang II‐induced podocyte cholesterol accumulation and injury.

## Graphical abstract

Ang II increases low‐density lipoprotein receptor (LDLR) abundance on the cell surface and causes podocyte cholesterol deposition: In addition to directly increasing LDLR expression, overactivation of Rab11‐mediated recycling and reuse of LDLR contributes to Ang II‐induced podocyte cholesterol accumulation and injury.

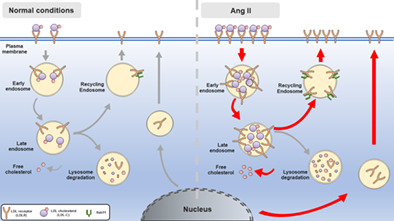

## 1 INTRODUCTION

The precise architecture of podocytes takes part in maintaining the selective permeability of the glomerular filtration barrier. For several decades, it has become clear that the occurrence of proteinuria in chronic kidney disease (CKD) is most often associated with foot process effacement and loss of podocytes.^1^ However, the cause of podocyte injury remains unrevealed. Cholesterol metabolism of podocytes is a complex process involving multiple metabolic pathways with points of regulation that are under genetic and metabolic controls.^2^, ^3^ Emerging evidence indicates that dysregulation of podocyte cholesterol has a central role in podocyte injury and might contribute to the development and/or progression of CKD.^4^, ^5^ Our previous studies demonstrated that angiotensin II (Ang II), a core effector of the activated renin‐angiotensin system (RAS), led to cholesterol accumulation in podocytes.^6^ However, the molecular mechanisms by which Ang II induced cholesterol accumulation in podocytes are not clear.

Of the many genes involved in cholesterol metabolism, we have recently reported the increased low‐density lipoprotein receptor (LDLR) expression in glomerular tissue obtained from kidney biopsies of patients with hypertensive nephropathy,^7^ as well as in the cultured podocytes with Ang II stimulation.^6^ Similarly, the upregulation of LDLR in the kidney has been reported in clinical and experimental diabetic kidney disease (DKD), leading to the influx of excess cholesterol into cells.^8^, ^9^ In fact, the abundance of LDLR is subjected to both transcriptional and non‐transcriptional controls. The precise regulation of genes mediating LDLR endocytosis, endocytic trafficking and degradation are closely associated with the required balance of LDLR at the cell surface and tightly regulated intracellular cholesterol metabolism.^10^, ^11^ The observation that LDLR siRNA in vitro is not sufficient to reverse cholesterol‐induced podocyte injury^8^ led us to hypothesise that post‐transcriptional regulation of LDLR may be a pivotal driver of cholesterol homeostasis in podocytes.

Rab11, a small GTPase and a member of the Rab superfamily, controls endocytic vesicular trafficking to the cell surface after sorting in recycling endosomes (REs).^12^ Recent evidence provides clues that Rab11 is involved in the regulation of LDLR trafficking,^13^ as well as the intracellular cholesterol distribution.^14^ However, whether altering the endocytic trafficking and degradation of LDLR via Rab11 would benefit podocytes from Ang II‐induced cholesterol dysregulation remains unknown. This study is aimed at investigating whether blocking LDLR recycling via Rab11 inhibition alleviates the effects of Ang II‐induced cholesterol accumulation and injury in podocytes.

## 2 MATERIALS AND METHODS

### 2.1 Animal studies

All experimental protocols were approved by the Animal Care Committee of Renmin Hospital of Wuhan University. An osmotic mini‐pump (Alzet model 2004) was implanted on the back between the shoulder blades in each of the male mice (C57BL6 background, 8 weeks of age), which were randomly assigned to receive the continuous infusion of saline (0.9% saltwater) or 700 ng/kg/min Ang II (Sigma‐Aldrich) for 8 weeks (another minipump was replaced after the fourth week). Twenty‐four hour urine was collected from metabolic cages every 2 weeks to measure the albumin‐to‐creatinine ratio (ACR). At the end of the experimental period, mice were sacrificed, and kidneys were isolated for glomeruli, or for pathological and biochemical assessments.

### 2.2 Podocyte culture

The *conditionally immortalised human podocytes* (HPCs) cell line was provided by Dr. Moin A. Saleem (Academic Renal Unit, Southmead Hospital, Bristol, UK). Podocytes were grown in RPMI culture medium (HyClone) containing 10% heat‐inactivated fetal bovine serum (FBS; Gibco), 100 U/ml penicillin G, 100 μg/ml streptomycin (Invitrogen), and 1× insulin–transferrin–selenium (ITS; Invitrogen) at 33°C for proliferation, then were shifted to 37°C for 14 days without ITS for differentiation. For Ang II stimulation, podocytes were treated with Ang II (10^−7^ M) for 24 h. For lysosome inhibition, podocytes were incubated with Leupeptin (20 mΜ, Topscience), an inhibitor of lysosomal protein degradation, for 30 min. For knockdown treatment, small interfering RNAs (siRNAs) targeting Rab11 (Qiagen) were transfected with HiPerFect (Qiagen) according to the manufacturer's instructions. For plasmid transfection, podocytes were transfected with the pEGFP‐Rab11a‐WT plasmid (Miaolingbio) using Lipofectamine 3000 Transfection Kit (Invitrogen) according to the manufacturer's instructions.

### 2.3 Antibody sources

Rabbit anti‐LDLR antibody (Invitrogen); mouse anti‐WT1 antibody (Novus); rabbit anti‐Rab11 antibody (Invitrogen) and mouse anti‐Synaptopodin antibody (Progen Biotechnik); mouse anti‐Lamp1 antibody (Progen Biotechnik); anti‐GAPDH mouse monoclonal antibody (Antgene); anti‐ATP1A1 rabbit polyclonal antibody (Proteintech); fluorescent secondary antibodies (Thermo Fisher Scientific).

### 2.4 Histological and immunohistochemical analyses

The pathological changes of glomeruli were evaluated by H&E staining. The glomerular sclerosis index^15^ was used to evaluate levels of glomerular sclerosis in each glomerulus as follows: 0, no sclerosis; 1+, 1–25%; 2+, 25–50%; 3+, 50–75% and 4+, 75–100%. Twenty glomeruli per mice (*n* = 5 per group) were assessed. Kidneys were fixed and the glomerular basement membrane and the fused foot processes were analysed by transmission electron microscopy. The foot process fusion rate was calculated as described.^16^

### 2.5 Quantitative reverse transcription‐PCR

Total RNA was extracted and reverse transcribed into cDNA as previously described. Quantitative reverse transcription‐PCR (qRT‐PCR) was performed using 50 ng cDNA and using primers specific for Rab11 and LDLR. The primers used are provided in the supplemental material.

### 2.6 Immunofluorescence assay

HPCs were seeded to glass slides, and the frozen kidney sections or slides were blocked and incubated overnight at 4°C with primary antibodies s (LDLR, Rab11, Lamp1), followed by incubation with fluorescent secondary antibodies at room temperature for 90 min in the dark. The nuclei were counterstained with 4′,6‐diamidino‐2‐phenylindole (DAPI, Antgene) for 5 min. All samples were recorded with a confocal laser scanning microscope (Olympus). Pearsonʼs coefficient and Mander's overlap coefficient indicate an actual overlap of the different signals and is considered to represent the degree of colocalization.^17^

### 2.7 Immunohistochemical assay

The paraffin‐embedded thick sections were deparaffinised, subjected to rehydration, antigen retrieval. Sections were blocked and incubated with primary antibodies overnight at 4°C. Then, the sections were incubated with HRP‐conjugated secondary antibody for 30 min. After washing with PBS, samples were treated with diaminobenzidine for 5 min and counterstained in haematoxylin. Slides were examined by microscope (Olympus), and representative images were shown.

### 2.8 Immunoprecipitation

Immunoprecipitation (IP) was carried out as described.^18^ Briefly, the total proteins were extracted using lysis buffer (Beyotime), and then incubated with protein A + G agarose suspension beads (Calbiochem) and Rab11 antibody overnight at 4°C with rotated. After being washed 3 times using lysis buffer, the beads were boiled in loading buffer at 100°C for 10 min. The samples were analysed by western blotting.

### 2.9 Western immunoblotting

Protein expression from podocytes or isolated glomeruli was measured by immunoblotting as previously described.^7^ The Membrane Protein Extraction Kit (Sangon Biotech) was used to extract membrane proteins according to the manufacturer's protocol. Briefly, samples were separated by SDS‐PAGE and then transferred to PVDF membranes (Millipore), and the membranes were blocked for 1 h at room temperature. The membranes were then incubated overnight at 4°C with a primary antibody. Next, the membranes were washed 3 times and labelled with a secondary antibody for 1 h in the dark. Protein band intensity was visualised by an LI‐COR Odyssey Infrared Imaging System. Quantitative analysis of protein bands was performed using Image J.

### 2.10 Flow cytometry

Apoptosis in cultured cells was determined by flow cytometry using annexin V‐FITC and 7‐ADD double staining according to the manufacturer's statement (BD).

### 2.11 Oil Red O staining

For histological visualization of total neutral lipids, kidney sections or cells on slides were fixed, rinsed with 60% isopropanol and incubated with 0.5% Oil Red O (Sigma‐Aldrich) working solution for 30 min. The slides were rinsed again with 60% isopropanol. Finally, the slides were counterstained with haematoxylin for 1 min and mounted. All slides were detected using an Olympus camera.

### 2.12 Filipin III staining

The cholesterol in the tissue or cells was stained by the cholesterol‐binding compound Filipin III (Cayman) for 2 h at room temperature. Fluorescence signals was analysed by a microscope (Olympus). Quantification of the staining was analysed by Image J.

### 2.13 Cholesterol quantification

The quantitative result of cellular cholesterol content was analysed by Cholesterol Assay Kit (Sigma‐Aldrich) and then measured using a microplate reader.

### 2.14 Statistics

For in‐vivo and in‐vitro experiments, representative results are shown. At least 3 experiments were conducted with 3–6 replicates per assay. Quantitative data were calculated as means ± SD, and statistical analyses were performed using GraphPad Prism 7.0 (GraphPad Prism Software, Inc.). Continuous variables for 2 groups were compared using Student *t*‐tests. Continuous variables for more than 2 groups were compared using 1‐way ANOVA test followed by Tukey's multiple comparison testing. *p* < 0.05 were considered as statistically significant.

## 3 RESULTS

### 3.1 Ang II infusion caused cholesterol dysmetabolism in podocytes

**FIGURE 1:**
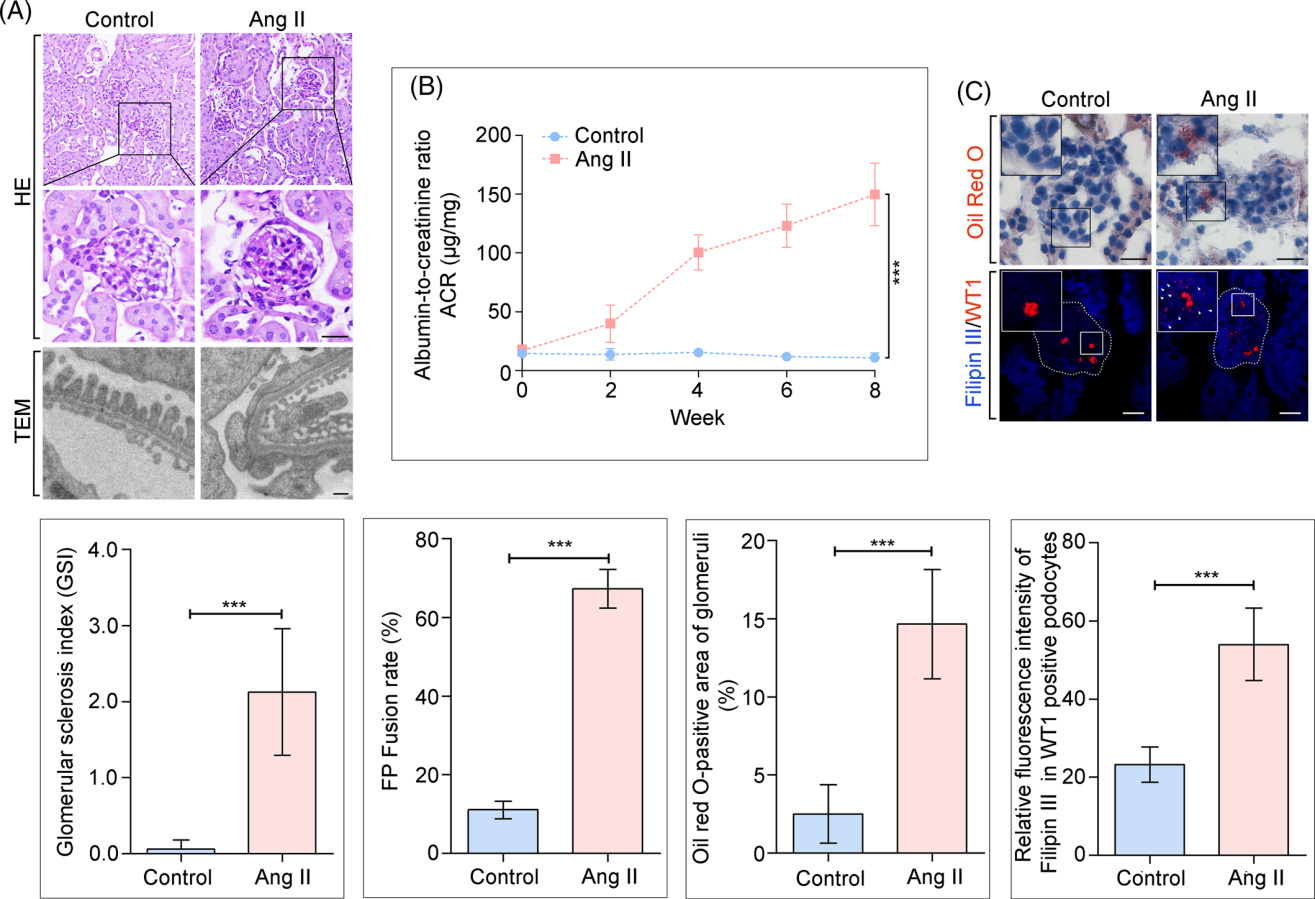
Ang II induced excessive glomerular cholesterol deposition and renal injury. Eight‐week‐old mice received a continuous infusion of Ang II for 8 weeks. Two groups of mice were analysed: Control (*n* = 5), Ang II (*n* = 5). (A) Representative pictures of H&E staining of renal cortical sections from different groups (scale bar: 20 μm), and bar graph analysis of the glomerular sclerosis index (GSI) to evaluate levels of glomerular sclerosis. Ultrastructure of the glomeruli by transmission electron microscopy (TEM) (scale bar: 1 μm), and bar graph analysis of the foot process fusion rate to evaluate levels of effacement; (B) Quantitative analysis of albumin‐to‐creatinine ratio (ACR) in different groups. ***p < 0.001, *n* = 5; (C) Representative microscopy images and quantification of lipid droplets by Oil red staining in kidney cortices (scale bar: 20 μm). Quantification of Filipin and WT1 (a marker of podocytes) double staining of kidney sections (original magnification ×600). ***p < 0.001, *n* = 30

We first constructed an Ang II infusion mouse model, consistent with our previous report,^7^ mice with Ang II infusion exhibited podocyte injury and renal dysfunction, as evidenced by a marked increase in glomerulosclerosis and mesangial matrix expansion (Figure 1A), exacerbated foot processes fusion (Figure 1A), and a significant ACR (Figure 1B) compared with the control group. To further assess the role of Ang II in podocyte cholesterol metabolism, we evaluated the lipid content in podocytes from mice with or without Ang II infusion. Neutral lipid staining by Oil red O revealed that Ang II infused mice had increased lipid droplets (LDs) in glomeruli (Figure 1C). Co‐labeling of Filipin III with WT1 (Wilms' tumour 1), a marker of podocytes, showed a marked accumulation of cholesterol in the glomeruli, especially in WT1‐positive podocytes from mice with Ang II infusion (Figure 1C). Similar findings were reported in previous studies on DKD,^9^, ^19^ Alport syndrome, and focal segmental glomerulosclerosis,^20^ indicating that renal cholesterol accumulation and lipotoxicity contribute to kidney dysfunction.

### 3.2 Changes in the expression of LDLR and Rab11 in podocytes with Ang II infusion

**FIGURE 2:**
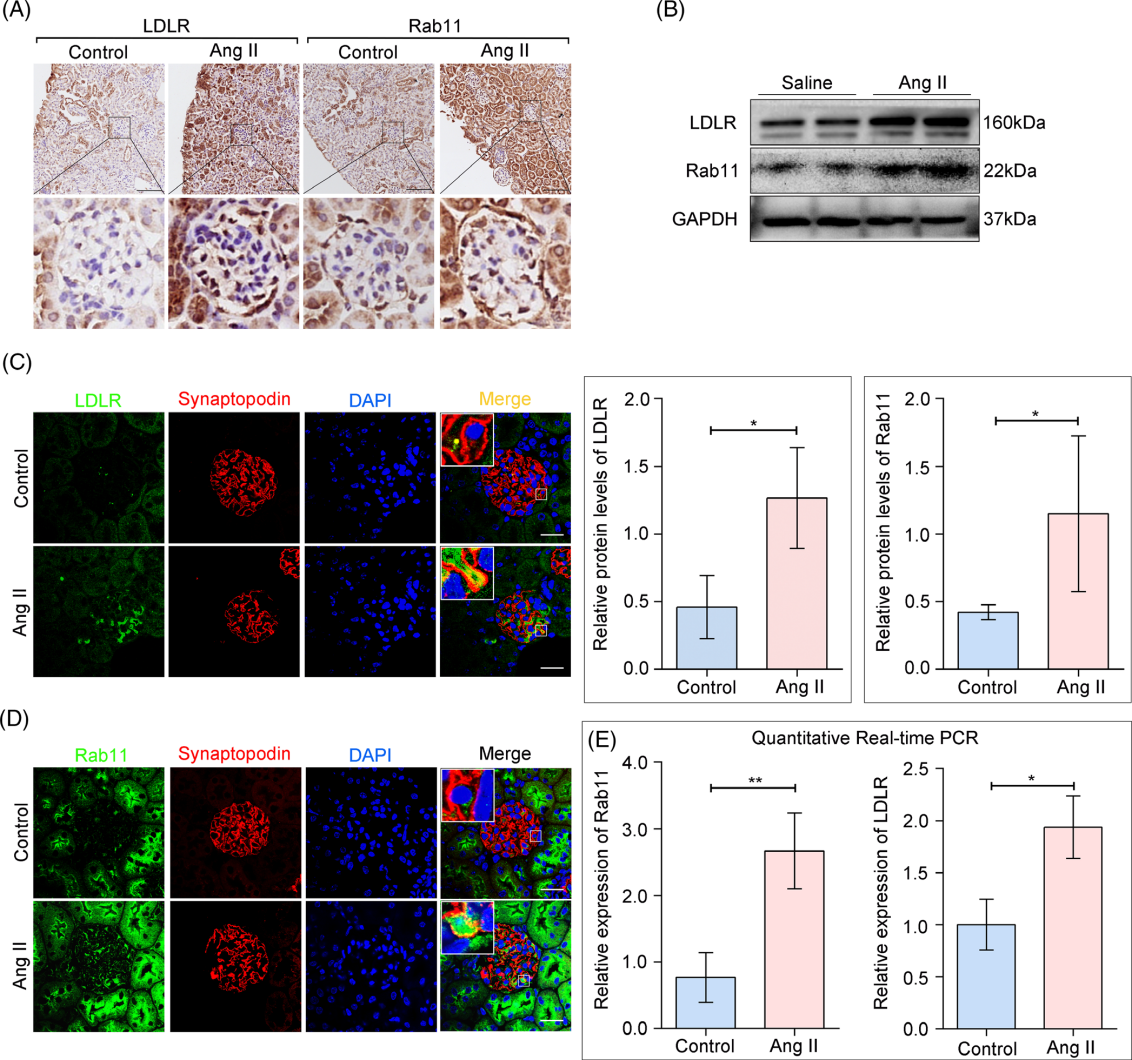
Expression alteration of LDLR and Rab11 in the glomeruli from Ang II‐infused mice. (A) Representative immunohistochemistry label of LDLR and Rab11 in kidney sections of control and Ang II infused mice. (original magnification ×40). (B) Western blot analysis of LDLR and Rab11 from glomerular lysates of control and Ang II treated mice. GAPDH was used as an equal loading marker, and the graph indicates a statistical result of relative protein levels. *p < 0.05, *n* = 5. (C and D) Fluorescence staining of LDLR or Rab11 (green) with Synaptopodin (a marker of podocytes, red), DAPI (nucleus, blue) to evaluate the expression levels (scale bar: 20 μm). (E) Quantitative RT‐PCR analysis to detect relative gene expressions in glomeruli from different groups. *p < 0.05, **p < 0.01, *n* = 5. DAPI, 4′,6‐diamidino‐2‐phenylindole; LDLR, low‐density lipoprotein receptor

We previously showed that LDLR could be upregulated by Ang II in podocytes in vitro.^6^ However, the specific mechanism of its non‐transcriptional regulation remains unclear. Since Rab11 has emerged as an important modulator of cholesterol metabolism through its regulation of recycling cargo,^14^ recent data suggest roles for Rab11 in the endocytosis and recycling of LDLR in a reversible manner.^13^ To verify whether Rab11/LDLR is involved in Ang II‐induced cholesterol metabolism disorder in podocytes, we then examined protein levels of LDLR and Rab11 in the glomeruli. Histochemical staining and Western blot analysis showed that there was a significant increase of LDLR and Rab11 in the glomeruli from Ang II infused mice (Figure 2A,B). Co‐staining of LDLR or Rab11 with Synaptopodin, a marker of podocytes, depicted increased protein levels of LDLR and Rab11 in podocytes from mice with Ang II infusion (Figure 2C,D). We also performed quantitative RT‐PCR and found a markedly elevated expression of LDLR and Rab11 in glomeruli from Ang II‐infused mice (Figure 2E). Consistent with our observations, a recent study has indicated that exposure to Ang II cause sustained activation of Rab11‐mediated endosomal recycling for its receptor AT1 (AT1R),^21^ led us to hypothesise that in addition to affecting LDLR expression, Ang II may also affect its content and distribution via Rab11‐mediated endosomal trafficking.

### 3.3 Ang II disrupted the interaction between LDLR and Rab11

**FIGURE 3:**
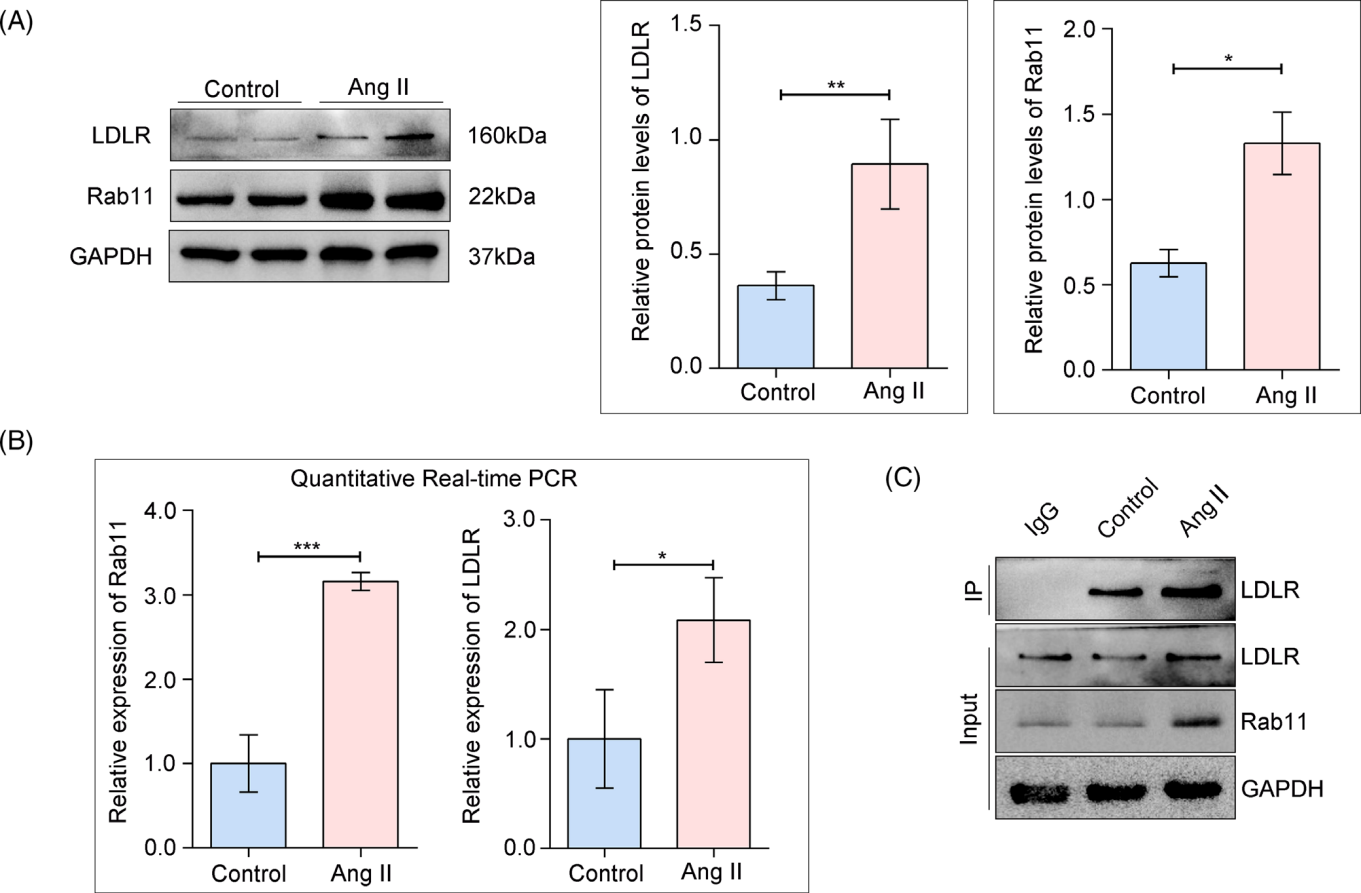
Enhanced interaction between LDLR and Rab11 in response to Ang II. (A) Western blot analysis of LDLR and Rab11 expression in HPCs exposed to Ang II, GAPDH was used as an equal loading marker, and the graph indicates a statistical result of relative protein levels. *p < 0.05, **p < 0.01, *n* = 5. (B) Quantitative RT‐PCR analysis to detect relative gene expressions in HPCs from different groups. *p < 0.05, ***p < 0.001, *n* = 5. (C) Co‐IP with anti‐Rab11 antibody or an IgG negative control. The resulting precipitates, as well as a portion of the whole cell lysate, were subjected to immunoblotting with anti‐LDLR, Anti‐Rab11, and anti‐GAPDH antibodies. HPCs, human podocytes; IP, immunoprecipitation; LDLR, low‐density lipoprotein receptor

To determine the role of Ang II in LDLR and Rab11 in vitro, HPCs were treated with Ang II. Western blots showed that both LDLR and Rab11 were significantly increased in HPCs stimulated with Ang II (Figure 3A). Likewise, elevated Rab11 and LDLR mRNA levels were demonstrated in podocytes with Ang II stimulation (Figure 3B). In addition, by performing co‐immunoprecipitation experiments, we revealed that LDLR was precipitated with Rab11 in HPCs, and the co‐precipitation effect was enhanced with Ang II stimulation (Figure 3C). Therefore, these data provide clues for the involvement of Rab11 in the intracellular trafficking of LDLR in podocytes.

### 3.4 Effects of Rab11 on the recycling and lysosomal degradation of LDLR

**FIGURE 4:**
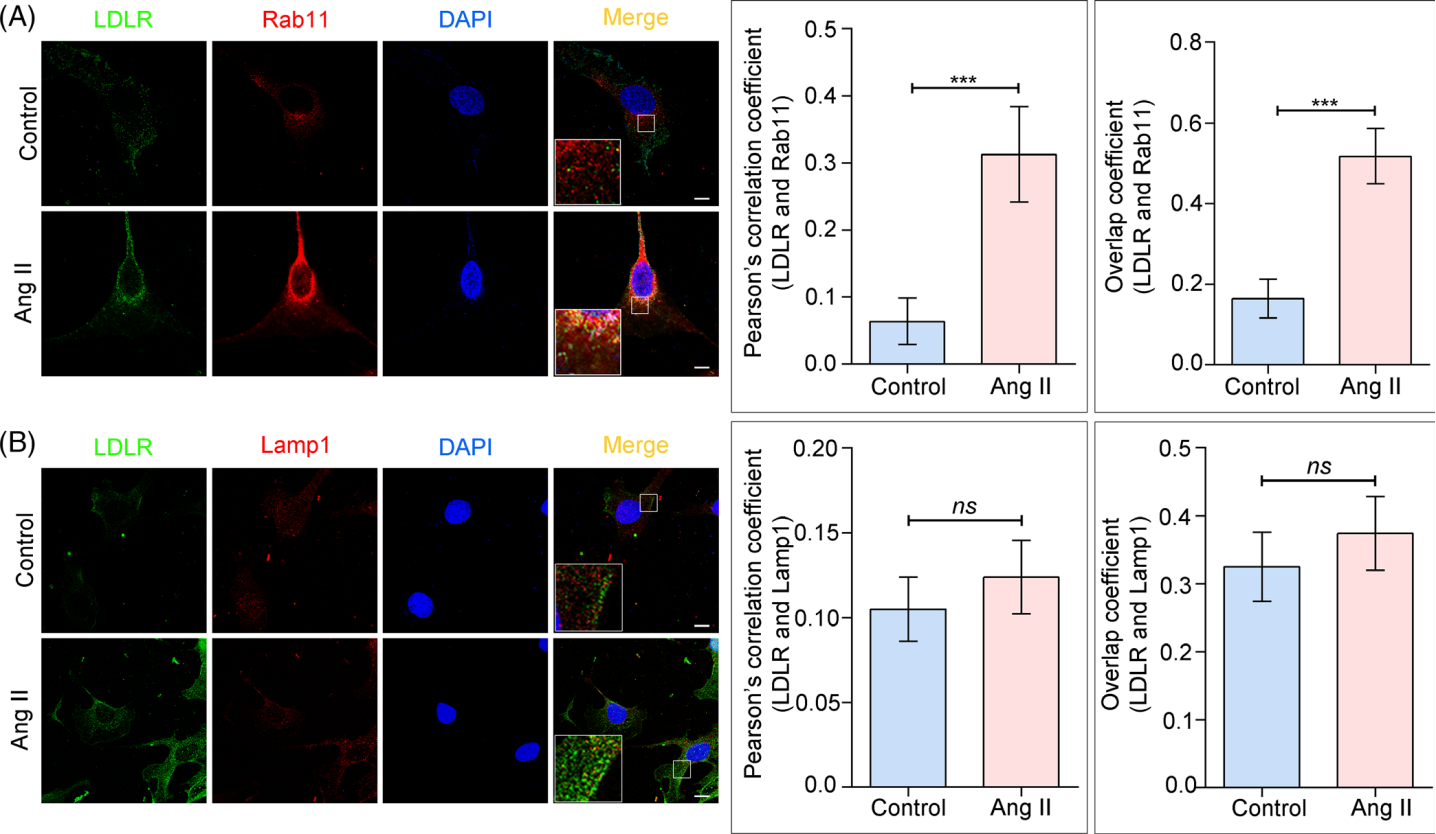
Ang II activated LDL recycling through Rab11. (A and B) Fluorescence staining of LDLR (green) with Rab11 (red) or Lamp1 (red) in HPCs (scale bar: 5 μm), and the graph indicates a statistical result of the average immunofluorescence intensity. Pearsonʼs coefficient and Mander's overlap coefficient are considered to represent the degree of panel colocalization. ***p < 0.001, ns = no significance, *n* = 30. HPCs, human podocytes; LDLR, low‐density lipoprotein receptor

Since the inactivation and clearance of LDLR depend on the lysosome‐dependent degradation,^22^ we next investigated the co‐localization of LDLR with Rab11 or the lysosome marker Lamp1 in vitro. Quantitation of the overlap of LDLR with Rab11 showed a considerable level of co‐localization after Ang II stimulation, as assessed by Pearson's correlation coefficient and the overlap coefficient (Figure 4A). Interestingly, Ang II stimulation did not affect the co‐location of LDLR and Lamp1 (Figure 4B). It is worth noting that receptor recycling is a highly effective process that actively opposes degradation, and a higher rate of LDLR recycling than degradation has been detected in previous studies.^23^ To further confirm the relationship between recycling trafficking and lysosomal degradation of LDLR, Leupeptin was applied as the lysosome inhibitor, and Western blot results showed that suppression of lysosomal degradation resulted in significant elevation of LDLR with or without Ang II exposure (Figure S1). Therefore, the upregulation of Rab11 in response to Ang II may further improve the recycling efficiency, while more LDLR were sorted towards the plasma membrane (PM) and less were accumulated in the endosome for degradation consequently.

### 3.5 Rab11 inhibition promoted the lysosomal degradation of LDLR

**FIGURE 5:**
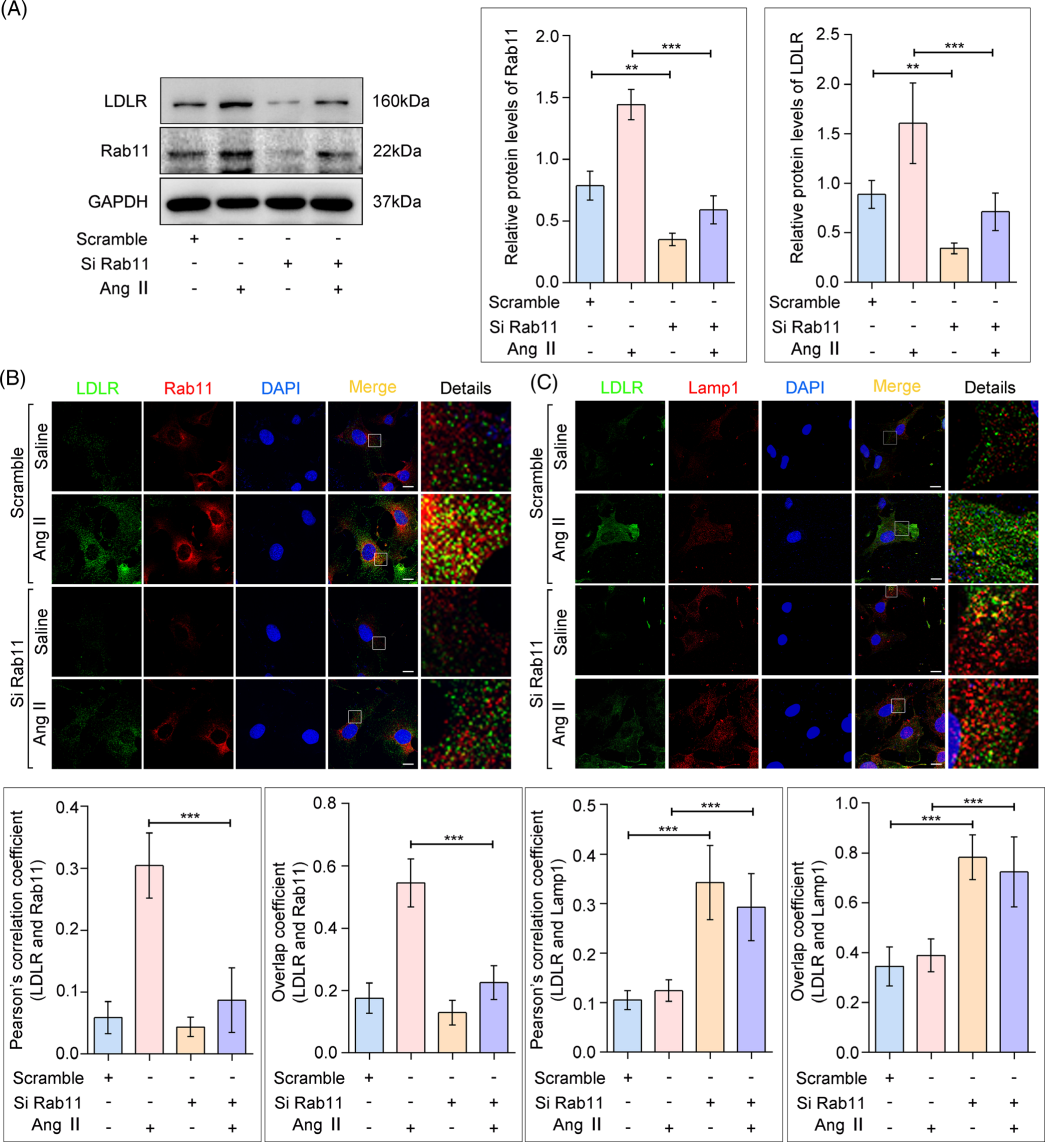
Rab11 knockdown reduced LDLR recycling and increased LDLR‐lysosomal degradation. HPCs were transfected with siRNAs targeting Rab11 (siRab11) or a scrambled control (Scramble) and then exposure to Ang II for 24 h. (A) Western blot analysis of total LDLR and Rab11 expression in HPCs, GAPDH was used as an equal loading marker, and the graph indicates a statistical result of relative protein levels. **p < 0.01, ***p < 0.001, *n* = 5. (B and C) Fluorescence staining of LDLR (green) with Rab11 (red) or Lamp1 (red) in HPCs (scale bar: 5 μm), quantification was performed as described in Figure 4. **p < 0.01, ***p < 0.001, *n* = 30. HPCs, human podocytes; LDLR, low‐density lipoprotein receptor

Next, we explored the mechanism underlying LDLR trafficking after the alteration of Rab11 expression. LDLR was detected by immunoblot analysis in total proteins and membrane proteins extracted from HPCs. As shown in Figure 5A and Figure S2A, transfection of small interfering RNA reduced the protein level of Rab11, resulted in a significant decrease of LDLR in both total and membrane proteins, and buffered the upregulation of LDLR caused by Ang II. In addition, the colocalization of Rab11 with LDLR in podocytes was reduced upon knockdown (Figure 5B). Interestingly, overexpression of Rab11 by plasmid transfection significantly increased LDLR expression (Figure S2B). Existing reports suggest that Rab11 plays a housekeeping role in regulating intracellular pathways between endocytic recycling, lysosomal degradation and exocytosis.^24^ Moreover, inhibition of Rab11‐ mediated reverse transport of cellular contents can promote the activation of its degradation through lysosome.^25^ In agreement, we observed that suppression of Rab11 promoted LDLR to enter the degradation pathway, manifested as more co‐localization of LDLR and Lamp1 (Figure 5C). These results highlight that Rab11 inhibition could suppress the protein level of LDLR, partially mediated by the activation of LDLR lysosomal degradation.

### 3.6 Inhibition of Rab11 decreased intracellular cholesterol accumulation and alleviated podocyte injury

**FIGURE 6:**
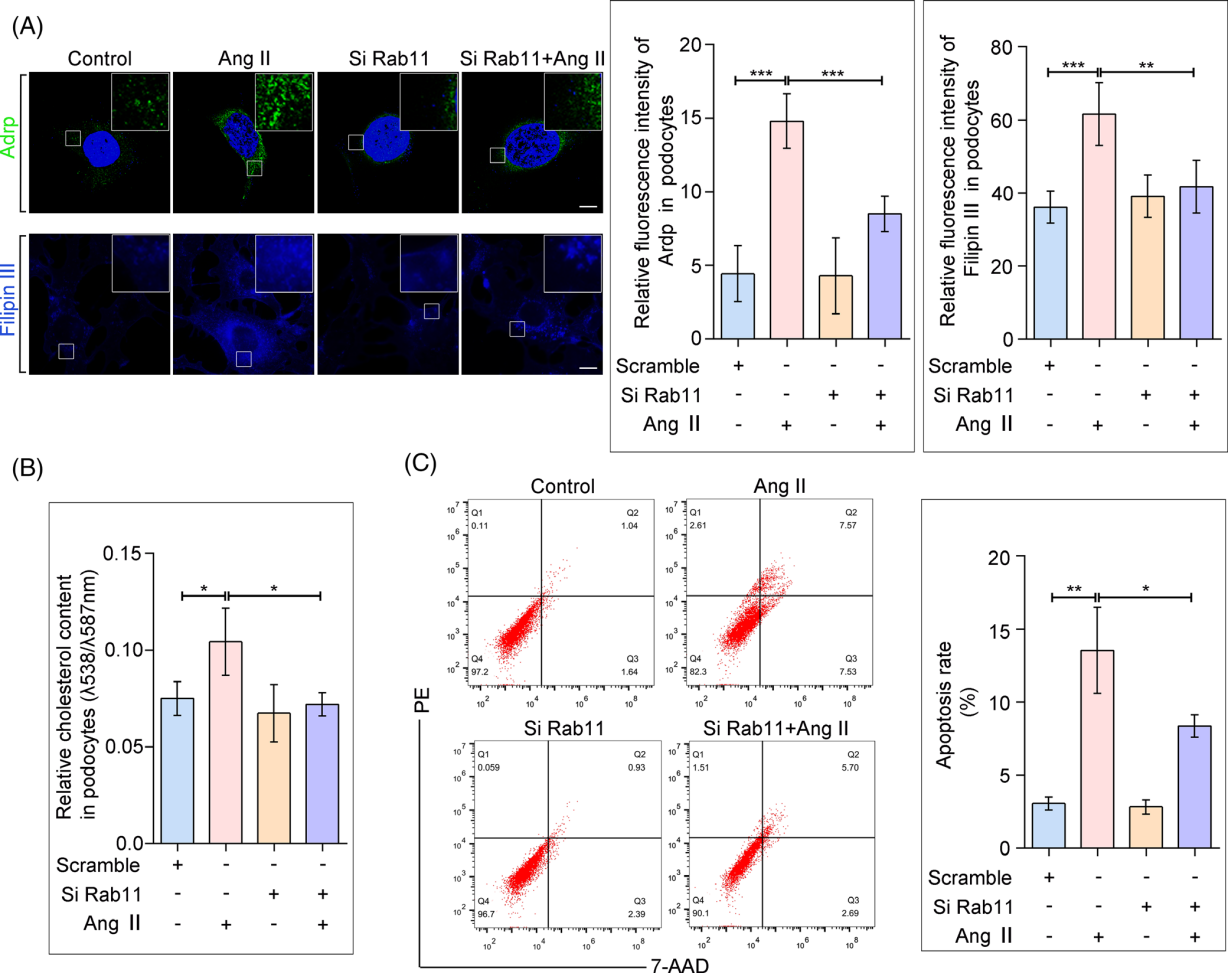
Rab11 inhibition reduced cholesterol and lipid droplets (LDs) content and protected podocytes from injury. (A) Representative confocal microscopy images and quantification of adipocyte differentiation‐related protein (Adrp, a marker of LDs) fluorescence staining and Filipin staining in each group. ***p < 0.001, *n* = 30. (B) Quantitative analysis of cholesterol content in each group. *p < 0.05, *n* = 6. (C) Flow cytometry analysis of podocytes apoptosis in different groups. *p < 0.05, **p < 0.01, *n* = 6

Lastly, to prove the potential regulatory role of Rab11 on podocyte cholesterol metabolism, we examined the content of LDs and cholesterol in vitro. As expected, we found that Rab11 siRNA treatment alleviated Ang II‐induced LDs and cholesterol accumulation (Figure 6A,B). However, overexpression of Rab11 further increased cholesterol content in podocytes (Figure S3). Of note, reports have implicated free cholesterol overload as an underlying metabolic factor, and exacerbated the pathology of cell injury through multiple pathways.^4^, ^26^ As the result shown, elevated intracellular cholesterol content exacerbated the apoptosis of podocytes, and the apoptosis rate of podocyte was rather reduced following Rab11 inhibition (Figure 6C). Collectively, these findings imply that Rab11 knockdown rescued the restraint of cell survival induced by cholesterol accumulation in podocytes, potentially via the inhibiting of LDLR recycling and further cholesterol influx.

## 4 DISCUSSION

In the present study, we have demonstrated for the first time that Ang II‐induced upregulation of Rab11 may contribute to cholesterol deposition in podocytes by increasing LDLR‐mediated cholesterol influx. We further define a new mechanism by which the recycling trafficking of LDLR is regulated by Rab11, inhibition of Rab11 increases lysosome‐mediated LDLR degradation, thus protecting podocytes from cholesterol accumulation and injury.

In recent years, massive lipid accumulation was detected in renal cells of CKD patients and animal models, and emerging evidence has indicated its cytotoxicity and adverse effects on renal function.^4^, ^9^, ^19^ Lipotoxicity is increasingly recognised as a leading cause of podocyte loss.^27^ However, the origin of cholesterol depositions in podocytes and its pathophysiological relevance remains enigmatic. Our recent study demonstrated that Ang II, a core effector of RAS activation, induced alteration in podocyte cholesterol homeostasis by disrupting the expression of genes involved in lipid metabolism, such as LDLR, ATP‐binding cassette A1 (ABCA1) and HMG‐CoA reductase.^6^, ^7^ Notably, cholesterol that enters podocytes originates mainly through LDLR. Cholesterol‐rich LDL particles bind to LDLR at the PM, together they are internalised via clathrin‐mediated endocytosis and further transported to early endosomes for dissociation.^28^ Although increased cholesterol influx of podocytes due to elevated LDLR expression has been reported in several pathological conditions, such as high glucose^8^ and inflammation,^29^ current studies mainly focus on its transcriptional regulation. In fact, LDLR levels on cell surfaces reflect a dynamic balance between transcriptional and post‐transcriptional processes. As a recycling receptor, internalised LDLR could be sorted towards different fates; the regulation between reuse and lysosomal degradation is central to maintaining its protein level.^30^, ^31^ Based on this, the inhibitors for proprotein convertase subtilisin/kexin 9 (PCSK9) are clinically applied to inhibit the degradation of LDLR in liver and used for cholesterol‐lowering therapies. However, PCSK9 dysfunction mutations are associated with increased circulating fasting glucose concentration, body weight and an increased risk of type 2 diabetes,^32^, ^33^ suggesting that changes in LDLR levels may trigger unknown metabolic changes for peripheral cells.

REs plays a critical role in the reuse of receptor molecules, so as in the remodelling of the protein and lipid composition of the PM.^34^ Specifically, for podocytes, we and others have found that appropriate recycling pathways are involved in endocytosis and recycling of slit diaphragm proteins, as well as cholesterol metabolism‐related proteins.^35^, ^36^ Here, we report the existence of increased LDLR abundance in podocytes in response to Ang II stimulation, partly due to Rab11‐mediated overactivation of the receptors recycling pathway.

Rab11 is one of the most extensively studied members of the Rab family and is known to function in endocytic membrane transport, including delivery of cellular components through REs and exocytosis.^12^, ^37^ In this study, we have demonstrated that treatment with Ang II upregulates the expression of Rab11 in podocytes. In line with our work, studies have proposed that Rab11 controls the endocytosis and recycling of Ang II type 1 receptor (AT1R), and the enhanced activity and upregulated expression of Rab11 are essential to maintain the continuous activation of RAS under pathological conditions.^21^, ^38^ Moreover, studies have found that Ang II involves in the activation of c‐JunN‐terminal kinase (JNK) signalling cascades.^39^ Interestingly, activation of the JNK/AP‐1 transcription factor pathway triggers the gene transcription of Rab11 and accelerates the receptors recycling.^40^ Therefore, it is conceivable that Ang II may promote Rab11‐mediated recycling of cargoes from REs to PM in podocytes by upregulation of Rab11 expression. In recent years, Rab11 has emerged as an important modulator of cholesterol metabolism, and particularly, its role in regulating the cholesterol content and distribution of organelles has been reported.^13^, ^14^ Interestingly, the overexpression of Rab11 resulted in an increase in dendritic branching in neurons,^41^ cell division and tumorigenesis in cancer,^42^ all of which requires an increased supply of cholesterol and other lipids for membrane proliferation. However, to date a role for Rab11 in cellular lipotoxicity has not been established. With this study, the enhanced binding of Rab11 to LDLR was found and further promotes the recycling/reuse of LDLR under Ang II stimulation, as a result, leading to increased podocyte cholesterol influx, which may reveal the mechanism by which Rab11 upregulation aggravates cholesterol deposition in cells.

As previously reported, receptor recycling is a highly effective and precise process that actively opposes degradation by directing internalised membrane proteins away from the degradation machinery.^34^, ^43^ Quantitative analysis of previous studies demonstrated a higher rate of that LDLR recycling than degradation in the physiological state, with each LDLR estimated to recycle 150 times.^23^ However, when the reuse of LDLR is blocked and accumulated in endosomes, it is more likely to be sorted towards the lysosome for degradation.^28^ Consistent with these findings, Leupeptin were applied to block the lysosomal degradation pathway, and we found that lysosome inhibition further increased the expression of LDLR in podocytes. However, we used siRNA to knockdown Rab11 and thus block the recycling of LDLR, resulting in an increase in lysosomal colocalization and degradation of LDLR. Consequently, reduced cholesterol deposition and palliative cell injury could be detected in podocytes.

Our study has several limitations: the in vivo evidence for the protection offered by Rab11 inhibition is lacking, and it is possible that other pathways are involved in the post‐transcriptional regulation of LDLR. The mechanism that Ang II may transcriptionally regulate Rab11 expression has not been thoroughly investigated. Besides, whether Rab11 regulates LDLR recycling through the GTPase activity remains unknown. In addition, the detection of renal histological specimens can further enhance the value of this study.

In conclusion, our studies demonstrated a novel function of Rab11 in determining the outcome of LDLR, which might be potentially vital for podocyte cholesterol metabolism. Rab11 inhibition protects podocytes from injury through the mechanism by the modulation of LDLR recycling and degradation, thus inhibiting LDLR‐mediated cholesterol influx. These findings may indicate that that post‐transcriptional regulation of LDLR may be a potential therapeutic strategy for CKD patients with altered renal cholesterol metabolism.

## CONFLICT OF INTERESTS

The authors declare that there is no conflict of interest.

## AUTHOR CONTRIBUTIONS

Jijia Hu, Zijing Zhu and Guohua Ding conceived and designed the experiments. Jijia Hu and Zijing Zhu performed the main experiments, analysed the data, and wrote the manuscript. Zhaowei Chen, Qian Yang and Wei Liang participated in some experiments. Guohua Ding revised the manuscript. All authors have read and approved the final manuscript.

## Supporting information


**FIGURE S1**Lysosome inhibition increased LDLR expression. Western blot analysis of total LDLR expression in HPCs, podocytes were incubated with Leupeptin (20 mΜ) for lysosome inhibition. GAPDH was used as an equal loading marker and the graph indicates a statistical result of relative protein levels. *p < 0.05, *n* = 5.

**FIGURE S2**Alteration in Rab11 expression affected total and membrane LDLR expression. (A) Western blot analysis of membrane LDLR in HPCs, ATP1A1 was used as an equal loading marker for membrane proteins and the graph indicates a statistical result of relative protein levels. **p < 0.01, *n* = 5. (B) Podocytes were transfected with the pEGFP‐Rab11a‐WT plasmid (Rab11 plasmid) or vehicle control (Vehicle) and then exposure to Ang II for 24 h. Western blot analysis of total LDLR and Rab11 expression in HPCs, GAPDH was used as an equal loading marker, the graph indicates a statistical result of relative protein levels. *p < 0.05, **p < 0.01, *n* = 5.

**FIGURE S3**Overexpression of Rab11 increased cholesterol and lipid droplets (LDs) content in podocytes. Representative confocal microscopy images and quantification of adipocyte differentiation‐related protein (Adrp, a marker of LDs) fluorescence staining, filipin staining in each group. *p < 0.05, ***p < 0.001, *n* = 30.

## Data Availability

Data supporting the findings of this study are available from the corresponding author upon reasonable request.
