# Supplementary figures and images for: Alteration in Rab11‐mediated endocytic trafficking of LDL receptor contributes to angiotensin II‐induced cholesterol accumulation and injury in podocytes
Source: Cell Prolif. 2022 May 14;55(6):e13229. doi: 10.1111/cpr.13229 (PMC9201372; doi:10.1111/cpr.13229)

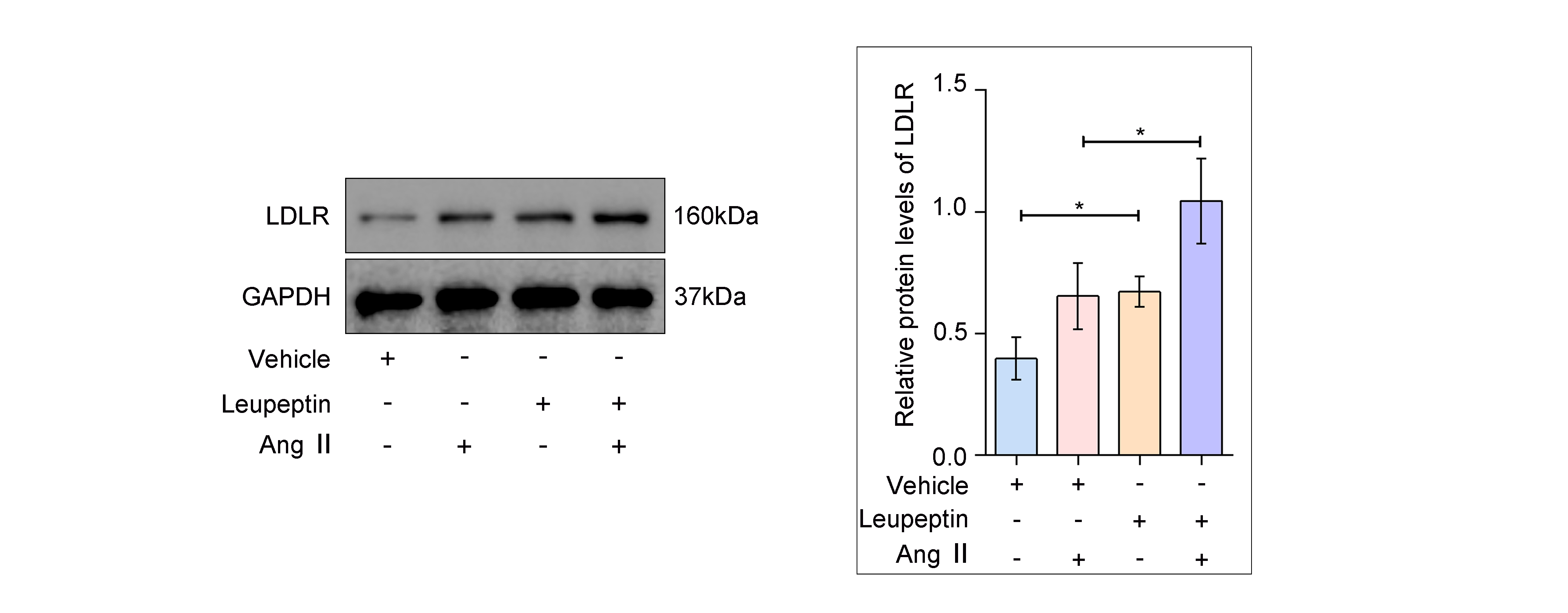

Supplement: Supplementary file 1 — FIGURE S1Lysosome inhibition increased LDLR expression. Western blot analysis of total LDLR expression in HPCs, podocytes were incubated with Leupeptin (20 mΜ) for lysosome inhibition. GAPDH was used as an equal loading marker and the graph indicates a statistical result of relative protein levels. *p < 0.05, n = 5. [file CPR-55-e13229-s001.tif]

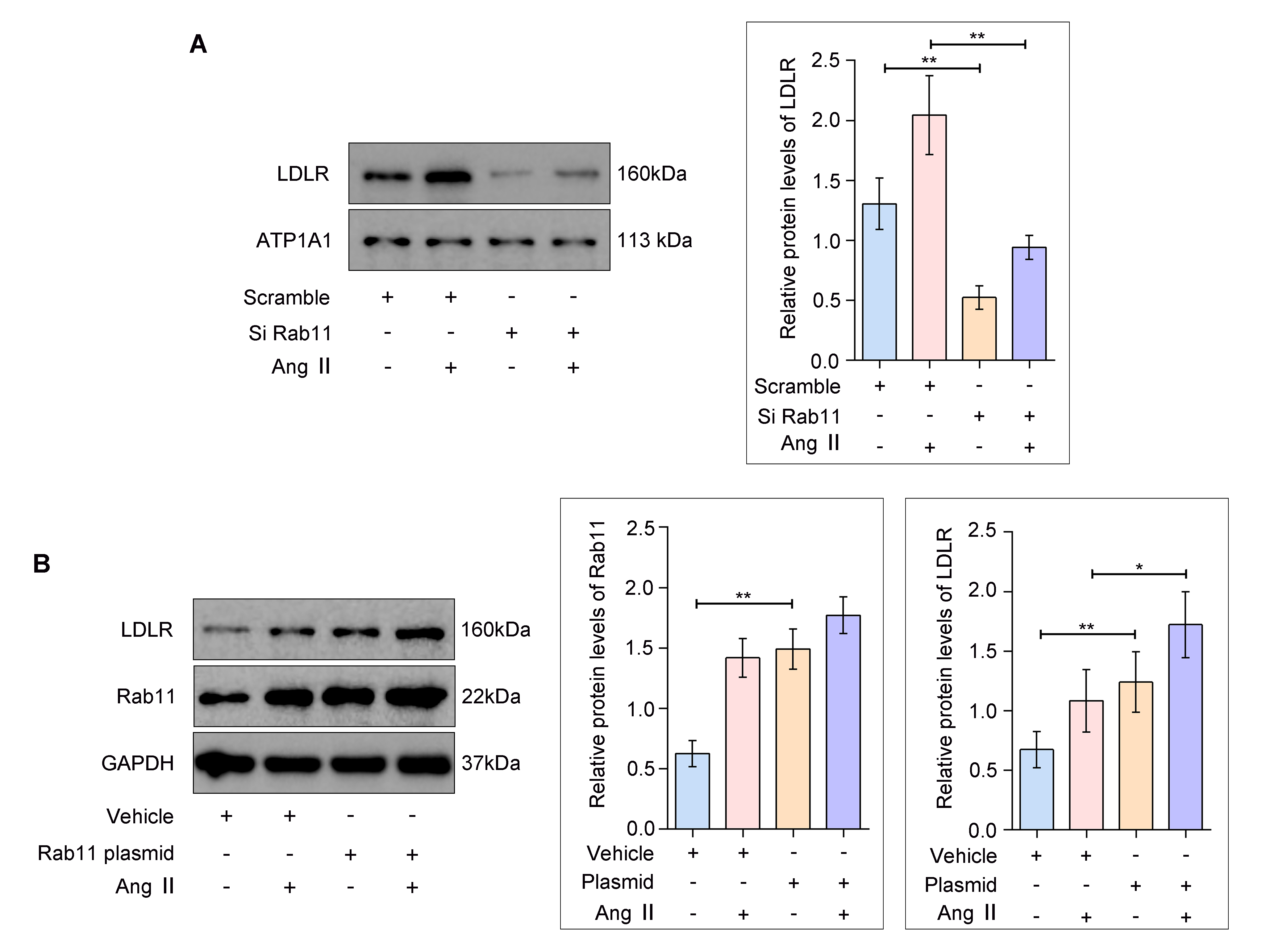

Supplement: Supplementary file 2 — FIGURE S2Alteration in Rab11 expression affected total and membrane LDLR expression. (A) Western blot analysis of membrane LDLR in HPCs, ATP1A1 was used as an equal loading marker for membrane proteins and the graph indicates a statistical result of relative protein levels. **p < 0.01, n = 5. (B) Podocytes were transfected with the pEGFP‐Rab11a‐WT plasmid (Rab11 plasmid) or vehicle control (Vehicle) and then exposure to Ang II for 24 h. Western blot analysis of total LDLR and Rab11 expression in HPCs, GAPDH was used as an equal loading marker, the graph indicates a statistical result of relative protein levels. *p < 0.05, **p < 0.01, n = 5. [file CPR-55-e13229-s002.tif]

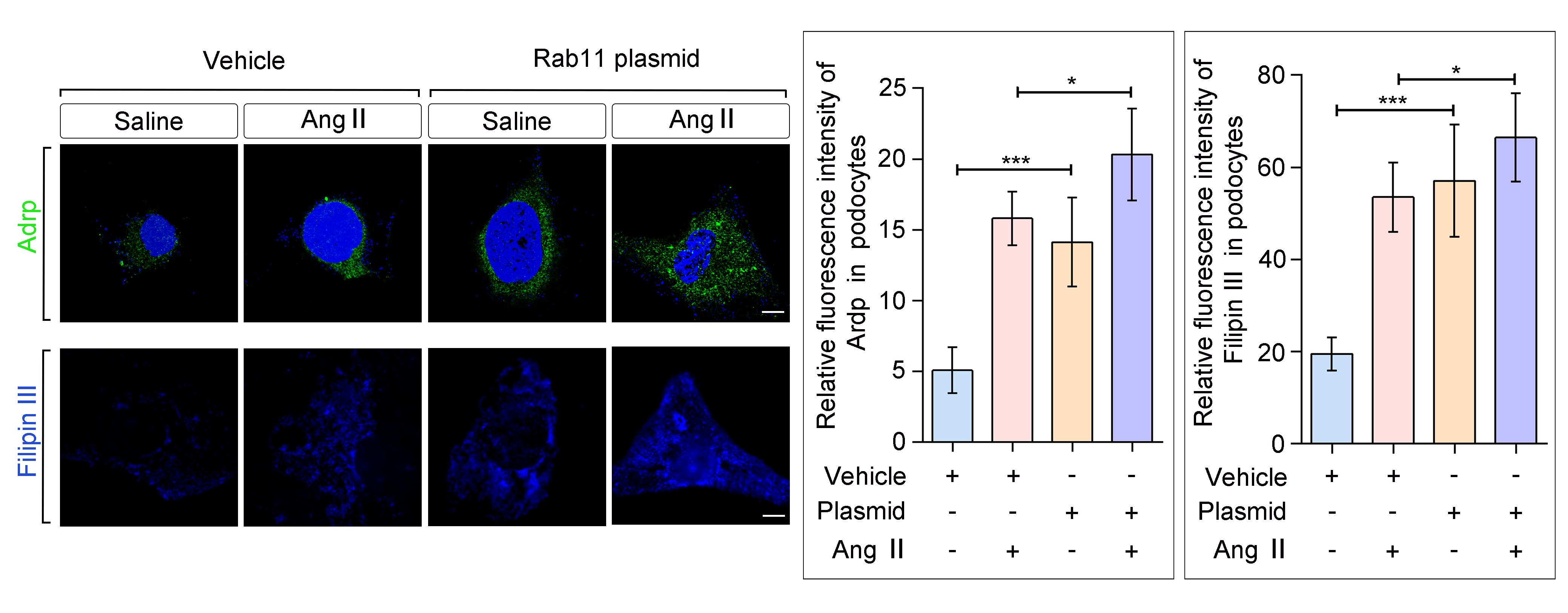

Supplement: Supplementary file 3 — FIGURE S3Overexpression of Rab11 increased cholesterol and lipid droplets (LDs) content in podocytes. Representative confocal microscopy images and quantification of adipocyte differentiation‐related protein (Adrp, a marker of LDs) fluorescence staining, filipin staining in each group. *p < 0.05, ***p < 0.001, n = 30. [file CPR-55-e13229-s003.tif]
